# Social Isolation and Anomalous Proprioceptive Experiences in Schizophrenia: A Case Study

**DOI:** 10.1192/bjo.2024.672

**Published:** 2024-08-01

**Authors:** Vullong Kwarkas, Geetha Jaganathan, Benjamin Cross

**Affiliations:** 1 Mersey Care NHS Foundation, Liverpool, United Kingdom; 2 Mersey Care NHS Foundation, St Helens, United Kingdom; 3 University of Liverpool, Liverpool, United Kingdom

## Abstract

**Aims:**

Integral to Bleuler's concept of schizophrenia, anomalous beliefs regarding the self are crucial to maladaptive social functioning. In schizophrenia, a predisposition to unusual bodily experiences, coupled with reduced awareness and increased social isolation, leads to hallucinations and delusions. Proprioceptive hallucinations, a subset of bodily hallucinations, present a challenging diagnosis due to their subjective nature, often resembling genuine bodily perceptions. We present the case of a 42-year-old man with untreated psychotic illness, manifesting perceptual abnormalities in the modality of proprioception.

**Methods:**

Mr. X was referred to Early Intervention in Psychosis (EIP), believing that all his joints were dislocated despite a normal neurological examination, Magnetic Resonance Imaging (MRI), and blood tests. Pertinently, childhood adversities and a seven-year history of prodromal and schizophrenia symptoms, chronic marijuana usage, potentially triggered by separating from his ex-partner, were present. At assessment, Mr. X recalled a delusional memory from age 5, seemingly heralding the onset of his illness. He displayed thought disorder, poor sleep and lacked insight. Olanzapine titrated to 15mg omni nocte (ON) improved sleep, but insight remained poor.

**Results:**

This case of rare proprioceptive hallucinations presenting in middle-age underscores the impact of positive schizophrenia symptoms on social impairment, suggesting a link between unusual bodily experiences and social isolation. Proprioception, encompassing joint perceptions, muscle force, and effort, contributes to body image by combining with exteroception. Interactions with others, influenced by our bodily sense, are crucial for adaptive social functioning. The social deafferentation hypothesis posits that loneliness in schizophrenia may heighten susceptibility to bodily aberrations. The psychological formulation and the chronic use of marijuana on Mr. X's psychopathology, although not thoroughly explored, cannot be overstated.

**Conclusion:**

Proprioception, vital for body image and social interactions, contributes to maladaptive functioning. The potent link between positive schizophrenia symptoms and social impairment needs exploring.

